# Assessing the Knowledge and Attitude towards Osteoporosis among Syrian Women: A Cross-Sectional Study

**DOI:** 10.1155/2022/6431151

**Published:** 2022-11-08

**Authors:** Ahmad Alhouri, Hanaa Zahrawi, Saja Alasaad, Shahd mofid Alhayek, Hasan Nabil Al Houri, Sami Jomaa, André Torbey, Sarya Swed, Douaa Alamash, Aous Zawda, Shahad Alhattab Alhasan, Naram Khalayli, Maysoun Kudsi

**Affiliations:** ^1^Faculty of Medicine, Syrian Private University, Damascus, Syria; ^2^Internal Medicine Department, Al Assad University Hospital and Al Mouwasat University Hospital, Damascus University, Damascus, Syria; ^3^Internal Medicine Department, Syrian Private University, Damascus, Syria; ^4^Faculty of Medicine, Damascus University, Damascus, Syria; ^5^Faculty of Medicine, Aleppo University, Aleppo, Syria; ^6^Rheumatology Department, Damascus University, Damascus, Syria

## Abstract

**Background:**

Osteoporosis is a progressive decline in the bone mass, which occurs with no alterations to the bone's composition. It is associated with increased bone fragility that may eventually lead to fractures. In this study, we aim to assess the level of awareness that Syrian women possess regarding osteoporosis and spread the knowledge about its prevention measures.

**Methods:**

This study was approved by the Institutional Review Board of the Syrian Private University. A validated questionnaire was asked to be filled in by women aged 18 and above, who were presented to clinics at Damascus, Al Helal, and Al Zahrawi hospitals between 28 November 2021 and 5 March 2022.

**Results:**

6082 women were included, of which 63.9% (*n* = 3884) were under 30 years old and 89.3% (*n* = 5429) were in their reproductive age. The average knowledge score of osteoporosis was 69.2 ± 7.7 (28-100); 88.1% correctly defined osteoporosis while the majority (93.4%) has heard of it. The majority of our participants were living in rural areas (77.2%; *n* = 4698) while only 22.8% (*n* = 1384) were living in urban areas. The respondents from urban areas reported the highest knowledge scores (OR = 1.472; 95% CI: 1.258-1.723; *P* < 0.0001). 75.60% agreed that aging is a risk factor for osteoporosis. 64.6% were aware that osteoporosis is directly responsible for hip fractures. Smoking, family history, lack of exercise, and menopause were the main risk factors for osteoporosis, at 53.6%, 53.1%, 84.6%, and 60.7%, respectively. Social media represented the ultimate source of information on osteoporosis (64.70%).

**Conclusion:**

Our study is the largest in the region and the first of its kind in the country. Syrian women had an average knowledge score regarding osteoporosis; the vast majority has defined it correctly and has heard of it. We found no statistical significance between age or educational level and adequate knowledge about osteoporosis.

## 1. Introduction

The worldwide prevalence of osteoporosis among elders is 21.7%, with the highest prevalence being in Asia (24.3%), followed by Europe (16.7%) and America (11.5%) [1]. Furthermore, the worldwide prevalence of osteoporosis in older women is 35.3% and 12.5% in older men [1]. Osteoporosis is a progressive decline in the bone mass, without alterations to the bone composition. It is associated with increased bone fragility that may eventually lead to fractures. The pathogenesis of osteoporosis is multifactorial and is affected by environmental, genetic, and hormonal influences [2]. Nonmodifiable risk factors for developing osteoporosis include increasing age, sex, premature menopause, previous fractures, parental hip fractures, and race. While the modifiable risk factors are low body mass index, smoking, and alcohol, as well as recent systemic corticosteroid use [3, 4]. Fractures may occur in any bone, but the most common sites are the hip and the spinal vertebrae [5]. The prevalence of osteoporosis in the Middle East was reported at 24.4% [1].

Saudi Arabia has the highest prevalence, with 32.7%, while Kuwait has the lowest with 15.1% [1]. A study from Syria reported that 36.84% of the studied women, which belonged to the age group between 50 and 59, were diagnosed with osteopenia in the lumbar spine and in the femoral neck in 68.42%, while osteoporosis was diagnosed in the lumbar spine in 23.68% of cases and 13.10% in the femoral neck [6]. 33.3% of women and 16.6% of men will sustain a hip fracture by their ninth decade [1]. Interestingly, 25% of hip fracture patients need institutionalization and over 30% will die within one year of the fracture [7]. The disease has a silent, progressive course that may present for the first time as a fracture without any previous complaints. Osteoporosis-related fractures are a real public health problem associated with significant morbidity, mortality, and economic burden, due to clinical consequences that impose substantial physical, psychosocial, and financial implications. Most women are probably unaware of the risk factors of osteoporosis, which play a crucial role in preventing its occurrence. Therefore, preventative measures such as patient education, as well as correcting misconceptions, can increase awareness about osteoporosis and consequently, reduce morbidity as well as mortality. In this study, we aim to assess the awareness level of Syrian women, regarding osteoporosis, and spread knowledge about its prevention methods.

## 2. Methods

This cross-sectional study was approved by the Institutional Review Board of the Syrian Private University (SPU); IRB 4-15-22. Informed consent was obtained from the patients who filled the questionnaire. Random sampling was used to collect data from major hospitals in the country. Between 28 November 2021 and 5 March 2022, women aged 18 and above, that were presented to clinics at Damascus, Al Hilal, and Al Zahrawi hospitals, were asked to fill in the validated questionnaire [8–10]. We used the Osteoporosis Knowledge Assessment Tool (OKAT), which was developed by Winzenberg et al. in 2003. OKAT was pretested and has been known to be valid and reliable [11–14]. OKAT was translated into Arabic by a specialized medical translator in order to verify the accuracy of the translation and to make sure that all respondents will comprehend the survey; 30 people were tasked with the responsibility of filling it out. The validity and reliability of the survey were then confirmed in a pilot test with 50 participants. Cronbach's alpha score for the utilize scale was above 0.4, which demonstrated that the tool maintained a strong internal consistency. The tool's reliability was reported previously; the internal consistency values were good for this instrument (Cronbach′s alpha = 0.824) [15]. Eight medical students distributed the survey in Arabic to women before their medical appointments. The questionnaire consisted of demographic and epidemiological data, reproductive status, risk factors, general questions about osteoporosis, sources of information on osteoporosis, and a specialized questionnaire to measure knowledge about the causes of osteoporosis, which was comprised of eighteen items rated on a 5-point Likert-type scale (strongly agree, agree, uncertain, disagree, and strongly disagree), as well as questions about osteoporosis prevention methods. The answers were evaluated immediately and discussed with the participating women to inform them about the current findings on osteoporosis and its preventative techniques.

### 2.1. Statistical Analysis

Data was analyzed using Statistical Package for Social Sciences (SPSS) version 28. Categorical variables were presented as frequency and percentages. In order to determine the differences between the menstrual status and the other variables that assess the knowledge of women about osteoporosis, we performed a Chi-squared *t*-test for the categorical variables and an Independent *t*-test for continuous variables. Binominal logistic regression was made to predict the presence of adequate awareness (the score > 75) of osteoporosis among Syrian women.

## 3. Results

### 3.1. Baseline Characteristics of the Study Sample

A total of 6082 Syrian women participated in the completion of the questionnaire. The majority of the participants were unmarried and under 30 years of age (Table 1). Subsequently, 77.2% of the women who participated were from rural areas and most had a moderate to good economic status. In addition, 5429 women were of reproductive age, representing 89.3% of the studied sample (Table 1).

### 3.2. Awareness towards Osteoporosis among Syrian Women

The participants' average knowledge score (KOS) on osteoporosis was 69.2225 ± 7.74803 (28-100). A substantial percentage of the women who took part in the study were familiar with osteoporosis as a disease (88.1%) and have heard of it (93.4%). Different percentages of women understood the risk factors of osteoporosis, including aging, female gender, race, smoking, sunlight, coffee intake, inheritance, and a small body frame, which were, respectively, 75.60%, 7.0%, 17.6%, 53.6%, 9.1%, 20.4%, 55.9%, and 18.02%. Only three percent of the participants had complete awareness of all foods that may help prevent the occurrence of osteoporosis. 60.7% of women are aware that menopause-related aging is an actual osteoporosis risk factor (Table 2).

### 3.3. The Difference in Awareness towards Osteoporosis among Syrian Women of Two Age Groups: The Reproductive Age Group and the Menopausal Age Group

A statistically significant difference was found when evaluating, having heard of osteoporosis as well as their understanding of the disease, between women in the reproductive and menopausal age groups, with women in the reproductive age group having had the highest percentages of awareness and understanding, with 85.1% and 97.4%, respectively (*P* value = 0.0001). Subsequently, there was a statistically significant difference in awareness regarding osteoporosis' risk factors, as women in the reproductive age group showed higher levels of awareness, including 5.7%, 47.3%, 7.3%, 17.2%, 76%, and 53.6% for the risk factors: female gender, smoking, tea drinking, coffee consumption, lack of exercise, and menopausal phase, respectively. In addition, we found a statistically significant difference (*P* value < 0.05) in the proper knowledge on osteoporosis prevention measures, between the two groups, with women in the reproductive age groups having had higher percentages of awareness about all of the mentioned preventative measures, including calcium-rich diet, walking to go shopping, and hormone treatment. Although the proportion of women in their reproductive age period who have had strong knowledge on the risks and preventative factors for osteoporosis is higher than that of the women in their menopause; the average knowledge score of osteoporosis was greater in the menopause age group, with the KOS in the reproductive and menopause age groups being 69 ± 7.6 and 71 ± 8.4, respectively (*P* value < 0.0001) (Table 3).

### 3.4. Predicted Factors of Adequate Knowledge towards Osteoporosis

The binominal logistic regression model was statistically significant, *X*^2^(19) = 469, *P* value < 0.001, and the Hosmer–Lemeshow test was 15. Of the variation in Syrian women's adequate understanding of osteoporosis (KOS > 75), this model predicted 11.8% (Nagelkerke R Square). Of the eight predictor variables, only four were statically significant: weight, height, the region, and the current menstrual status (as shown in Table 4). Women who had weights between 50-80 kg had 3.6-4.2 times higher odds of adequate knowledge compared to those who had weights less than 50 kg. Also, the respondents in the city (vs. rural regions, OR = 1.472, 95% (CI: 1.258-1.723), *P* < 0.0001) obtained a higher knowledge score. Eventually, women within the menopausal period had 1.381 times higher odds of adequate knowledge compared to women of reproductive age, with a confidence interval between 1.022 and 1.865 and a *P* value = 0.036.

### 3.5. The Source of Information on the Osteoporosis

**Figure 1:**
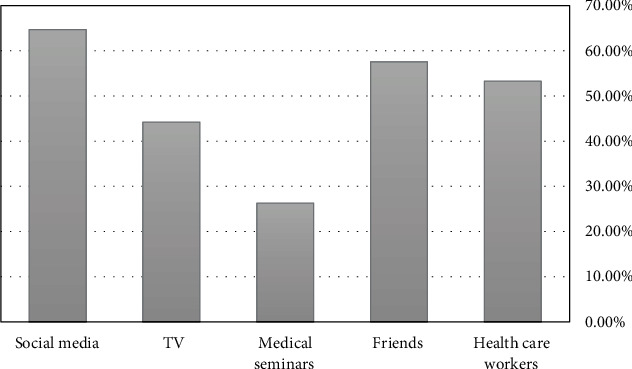
The source information on osteoporosis among the participating women.

Social media was the approach that was utilized the most as a source of information on osteoporosis among the Syrian women population. As compared to medical seminars, which had a usage rate of 26.30, social media had a usage rate of 64.70% (Figure 1).

## 4. Discussion

Our study is the first and the largest to assess knowledge and awareness among Syrian women (*n* = 6082) about osteoporosis. The main findings of our study are as follows. First, the vast majority has heard or was familiar with the disease, with an average knowledge score of 69.2 ± 7.7; however, the average score was significantly higher in women in the menopausal age group (71 ± 8.4 vs. 69 ± 7.6 for women of reproductive age). Secondly, women in the reproductive age group had the highest percentages of awareness and understanding of osteoporosis, its risk factors, and its preventative measures (calcium-rich diet, walking to go shopping, and hormone treatment). In addition, more than half of the participating women were aware that menopause-related aging is an actual osteoporosis risk factor. Finally, social media was the most common source of information on osteoporosis among the Syrian women population.

The prevalence of osteoporosis in China increased from 20% in 2016 to 39.4% in 2020 [16, 17]. The National Osteoporosis Foundation anticipated that by 2020, osteoporosis will affect more than 60 million Americans aged ≥50 [18]. In a recent systematic review and meta-analysis, the overall global prevalence of osteoporosis was reported to be 35.3% in older women and 12.5% in older men. In addition, Asia had the highest prevalence of osteoporosis among the elderly (24.3%) [1]. It was estimated that 1 in 3 women after the age of 50 would experience osteoporosis-related fractures [16, 19, 20]. Therefore, increasing awareness among the general population and establishing long-term preventative strategies evolved as mandatory demands to decrease the burden on the health system [21, 22].

Our study included 6082 women, of which 63.9% (*n* = 3884) were under 30 years of age and 89.3% (*n* = 5429) were of reproductive age. The average knowledge score on osteoporosis was 69.2 ± 7.7 (28-100); 88.1% defined osteoporosis correctly while the majority (93.4%) has heard of it. A Vietnamese study by Nguyen et al. reported an awareness rate of 82% [23], while a study from Taiwan reported that only 49.5% of women had some knowledge of osteoporosis [24], which is considered relatively low. A Turkish study which included 768 women aged between 40–70 years, distributed across three rural towns, used a 20-item questionnaire to measure their knowledge on osteoporosis. They found that 60.8% of participants have heard of osteoporosis while only 44.9% have defined it correctly [21]. A quasiexperimental study was conducted in Jordan to measure the knowledge regarding osteoporosis among 148 female students, aged between 16 and 18 years [25]. They assessed their knowledge before and after a series of educational sessions and found a significant overall increase in the osteoporosis knowledge mean scores from 24.1 to 29.8. However, they found no changes in scores related to fracture risk factors, osteoporosis diagnosis, treatment of osteoporosis, and fracture pain. Therefore, further educational sessions were suggested. Another study from South Asia reported similar results [26].

Many studies have addressed the knowledge differences between urban and rural areas [16, 21, 27]. The majority of our participants were living in rural areas (77.2%; *n* = 4698) while only 22.8% (*n* = 1384) were living in urban areas. The respondents from urban areas reported higher knowledge scores (OR = 1.472; 95% CI: 1.258-1.723; *P* < 0.0001). A systematic review and meta-analysis form China [16] reported a higher, but not significant, prevalence of osteoporosis among rural residents (23.92% vs. 20.87% in urban residents). Sitati et al. [27] used the Facts on Osteoporosis Quiz (FOOQ) scale to measure knowledge in Kenyan semirural county of Kiambu. 254 African postmenopausal women aged ≥50 were included. They reported a mean score of 8.6 out of 17, which also reveals poor knowledge among rural residents. The Turkish study also reported an average score of 5.52 out of 20 among rural areas [21]. It was suggested that the declining levels of socioeconomic status were a major factor for decreased awareness levels of osteoporosis [27].

Our study revealed that 60.7% of women recognized menopause-related aging as a risk factor for osteoporosis and 75.60% agreed that aging is a risk factor for osteoporosis. In addition, 64.6% of the total sample was aware that osteoporosis is directly responsible for hip fractures. Many studies found that women of younger ages and advanced educational levels defined osteoporosis correctly compared to older and less educated ones [21, 22, 28–33]. A cross-sectional study from Lebanon that included 560 women aged ≥40 years reported a relatively low knowledge score among participants (47.3%) and stated that low educational levels and a lack of adequate exercise were associated with lower knowledge scores [34]. A cross-sectional study used Osteoporosis Prevention and Awareness Tool to measure the awareness regarding osteoporosis among Singaporean women and reported that 88.3% (*n* = 362) had low scores; moreover, these scores were reported in women with older ages and/or lower educational levels [31]. Another Malaysian study used the same tool to assess knowledge among 284 community pharmacists and reported a relatively high score, with a mean of 10.1 ± 3.4 out of 15 risk factors [35]. On the other hand, two studies from Singapore [31] and Canada [36] found that women's age had no significant association with osteoporosis knowledge. However, we found no statistical significance between age or educational level and adequate knowledge on osteoporosis; this could be explained by the fact that 63.9% of our sample was under 30 years of age.

Our study found that smoking, family history, lack of exercise, and menopause were the main risk factors for osteoporosis, 53.6%, 53.1%, 84.6%, and 60.7%, respectively. In addition, 36.9% of women in our sample believed that calcium-rich diets have a protective effect against osteoporosis. Although some variations in the percentage of each factor may exist, these results and factors are consistent with previously published studies [21, 22, 28, 30, 33]. A recently published Jordanian study reported that older ages, being physically inactive and being less sun-exposed, were major risk factors whereas educational level had no significant association with osteoporosis [37].

Social media represented the ultimate source of information on osteoporosis among Syrian women (64.70%). A study from Lebanon revealed that television and the Internet were the most common sources of knowledge [34]. The Turkish study reported that 55% of women stated that television is the leading source of knowledge. On the other hand, they found that women ≥60 years of age considered doctors as the first source of information. They suggested that the increased incidence of diseases in this age group could explain these results [21]. A Saudi study reported that students' friends were the core source of information (32.1%). In addition, 83.1% of students deemed that the healthcare providers do not deliver sufficient information regarding osteoporosis [22]. A study from Thailand reported similar results [24]. A Singaporean study revealed that television and healthcare providers were the most common sources of osteoporosis-related information, 34.9% and 30.7%, respectively [31].

Our study is the first and the largest to assess knowledge and awareness about osteoporosis among Syrian women. It provides a comprehensive picture regarding the socioeconomic and educational status and how it might impact the health sector. In addition, it provides an epidemiological background to implement educational purposes and preventive actions in the aim of lowering the prevalence of osteoporosis in Syria. However, our study has its limitations, as it was designed as a cross-sectional one. In addition, the majority of our respondents were ≤30 years old and included only women participants. Both of these factors may create some bias. Another limitation is that the majority of our studied sample was in the reproductive age group, which causes some bias and makes the results less generalizable. Although, we enrolled every patient during the study period that was older than 18 years and we did not exclude patients based on their reproductive status. Therefore, no specific reason for this limitation was determined.

## 5. Conclusion

Our study is the largest study in the region and the first of its kind in the country. Syrian women had an average knowledge score on osteoporosis, and the vast majority has defined osteoporosis correctly and has heard of it, which is a positive indicator of a good health education system. However, we found no statistical significance between age or educational levels and adequate knowledge on osteoporosis.

## Figures and Tables

**Table 1:** Demographic variables of the study sample.

Variables	Categories	Frequency	Percentage
Age	18-30	3884	63.9%
	31-40	1058	17.4%
	Above 40 years	1140	18.7%

Social status	Single	3431	56.4%
	Married	2271	37.3%
	Divorced	157	2.6%
	Widow	223	3.7%

Weight	Under 50 kg	695	11.4%
	50-60 kg	2307	37.9%
	61-80 kg	2538	41.7%
	Above 80 kg	464	7.6%
	Prefer to not say	78	1.3%

Height	Under 150 cm	63	1.0%
	150-160 cm	2603	42.8%
	161-170 cm	2844	46.8%
	Above 170 cm	390	6.4%
	Prefer to not say	182	3.0%

Nationality	Syrian	5387	88.6%
	Non-Syrian	695	11.4%

Region	City	1384	22.8%
	Rural region	4698	77.2%

Unhealthy habits practice daily	Smoking	459	7.5%
	Hookahs	1363	22.4%
	Both of them	192	3.2%
	Alcohol	58	1.0%
	All the above	157	2.6%
	None of the above	3852	63.3%

Educational level	Illiterate	94	1.5%
	Primary school	192	3.2%
	Secondary school	750	12.3%
	University stage or above	4723	83%

Economic level	Bad	250	4.3%
	Moderate	1257	20.7%
	Good	4168	68.5%
	High	397	6.5%

Menstrual status	Age of reproductive activity (existing menstrual cycle)	5429	89.3%
	Menopause period (menstrual cycle stopped)	653	10.7%

Working status	Full time work	1093	18.0%
	Partial time work	880	14.5%
	I am student and worker	547	9.0%
	I do not work because I am a student	1627	26.8%
	I do not work because I am a housewife	1935	31.8%

**Table 2:** Descriptive table of the questionnaire used to assess knowledge on osteoporosis among Syrian women.

Questionnaire item		Strongly agree	Agree	Uncertain	Disagree	Strongly disagree	Positive answers %
1	Have you heard about osteoporosis? If yes from whom?						93.4% (yes)
2	What is osteoporosis? (bone loss, melting, etc.)						88.10%
3	Osteoporosis is directly responsible for disabling hip fracture.	10.30%	54.30%	22.00%	10.80%	2.60%	64.60%
4	A potential outcome of the disease is death.	0.70%	7.10%	19.40%	53.00%	19.80%	7.80%
5	Aging is a risk factor for osteoporosis.	12.90%	62.70%	13.40%	8.10%	2.90%	75.60%
6	Male gender is a risk factor for osteoporosis	0.60%	6.40%	34.60%	44.30%	14.00%	7%
7	Race is a risk factor for osteoporosis.	1.80%	15.80%	39.20%	33.00%	10.10%	17.60%
8	Smoking may be a risk factor for osteoporosis.	9.50%	44.10%	26.70%	15.30%	4.50%	53.6%
9	Sunlight is a risk factor for osteoporosis.	1.90%	7.20%	11.00%	35.60%	44.40%	9.10%
10	Tea consumption may be protective against osteoporosis.	0.90%	7.00%	28.40%	43.30%	20.40%	1.60%
11	Coffee consumption may be a risk factor for osteoporosis.	1.90%	18.50%	39.30%	32.80%	7.60%	20.40%
12	Heredity may be a risk factor for osteoporosis.	7.60%	48.30%	25.20%	14.80%	4.10%	55.90%
13	Small body frame may be a risk factor for osteoporosis.	1.80%	16.30%	35.40%	37.00%	9.50%	18.10%
14	If your first-degree relatives have osteoporosis, you may also have a risk.	11.60%	41.50%	29.00%	13.90%	3.90%	53.10%
15	A calcium-rich diet has a protective effect against osteoporosis.	3.20%	33. 7%	31.80%	24.40%	6.90%	36.90%
16	Lack of exercise may be a risk factor for osteoporosis.	23.90%	60.70%	7.40%	3.80%	4.20%	84.60%
17	Menopause is a risk factor for osteoporosis.	9.10%	51.60%	23.30%	11.90%	4.20%	60.70%
18	Some medications may be a risk factor for osteoporosis.	11.90%	55.50%	22.60%	6.80%	3.20%	67.40%
19	Walking to go shopping will protect you from osteoporosis.	3.50%	34.60%	39.40%	18.50%	4.00%	38.10%
20	A salty diet may cause you to lose calcium.	2.90%	23.20%	50.60%	19.20%	4.10%	26.10%
21	Have you ever talked to your physician about osteoporosis?						Yes: 11.3%
22	Would you be interested in discussing osteoporosis with your physician? If not, why?						Yes: 66.6%
23	Which of the following are correct for osteoporosis? (a) Prevention is impossible (b) There is no treatment (c) A calcium-rich diet is essential for prevention (d) Calcium supplements may be used in addition to dietary calcium (e) Vitamin D is also recommended in prevention (f) Female hormones (estrogens) may be used for prevention						All true: 24.4%
24	Imagine that you are told that you have osteoporosis or you are just at the beginning of the menopause. Would you agree to use hormone replacement therapy?						Yes: 25.3%
25	Which of the following may be preventive against osteoporosis when consumed regularly and in adequate amounts? (a) Milk, (b) cheese, (c) meat, (d) coffee, (e) yoghurt, (f) sugar, (g) olive oil, (h) margarine, or (i) fruit and vegetables.						All true: 3%
26	If we offer an osteoporosis public seminar, would you like to attend? If yes, what should be the duration of the seminar?						Yes: 92.2%

**Table 3:** The differences in awareness towards osteoporosis, between women in the reproductive age group and the menopausal age group.

Questionnaire item		Age of reproductive activity	Menopause period	*P* value
1	Have you heard about osteoporosis? If yes from whom?	85.1%	8.40%	<0.0001^a^
2	What is osteoporosis?	79.4%	8.70%	<0.0001^a^
3	Osteoporosis is directly responsible for disabling hip fracture.	57%	7.60%	<0.0001^a^
4	A potential outcome of the disease is death.	6.80%	1%	0.064^a^
5	Aging is a risk factor for osteoporosis.	68%	7.70%	0.11^a^
6	Male gender is a risk factor for osteoporosis.	5.70%	1.40%	<0.0001^a^
7	Race is a risk factor for osteoporosis.	15.60%	2%	0.38^a^
8	Smoking may be a risk factor for osteoporosis.	47.30%	6.20%	0.01^a^
9	Sunlight is a risk factor for osteoporosis.	8.20%	0.90%	0.37^a^
10	Tea consumption may be protective against osteoporosis.	7.30%	0.60%	0.054^a^
11	Coffee consumption may be a risk factor for osteoporosis.	17.20%	3.20%	<0.0001^a^
12	Heredity may be a risk factor for osteoporosis.	49.90%	6%	0.8^a^
13	Small body frame may be a risk factor for osteoporosis.	16.90%	1.30%	<0.0001^a^
14	If your first-degree relatives have osteoporosis, you may also have a risk.	45.40%	7.80%	<0.0001^a^
15	A calcium-rich diet has a protective effect against osteoporosis.	32.60%	4.40%	0.04^a^
16	Lack of exercise may be a risk factor for osteoporosis.	76%	8%	<0.0001^a^
17	Menopause is a risk factor for osteoporosis.	53.60%	7.10%	0.003^a^
18	Some medications may be a risk factor for osteoporosis.	59.70%	7.70%	0.016^a^
19	Walking to go shopping will protect you from osteoporosis.	33%	5.10%	<0.0001^a^
20	A salty diet may cause you to lose calcium.	22.60%	3.50%	<0.0001^a^
21	Have you ever talked to your physician about osteoporosis?	7.90%	3.40%	<0.0001^a^
22	Would you be interested in discussing osteoporosis with your physician?	58.60%	8%	<0.0001^a^
23	Which of the following are correct for osteoporosis? (a) Prevention is impossible (b) There is no treatment (c) A calcium-rich diet is essential for prevention (d) Calcium supplements may be used in addition to dietary calcium (e) Vitamin D is also recommended in prevention (f) Female hormones (estrogens) may be used for prevention.	18.80%	5.70%	<0.0001^a^
24	Would you agree to use hormone replacement therapy?	3.10%	4.20%	<0.0001^a^
25	Which of the following may be preventive against osteoporosis when consumed regularly and in adequate amounts? (a) Milk, (b) cheese, (c) meat, (d) coffee, (e) yoghurt, (f) sugar, (g) olive oil, (h) margarine, or (i) fruit and vegetables.	2.80%	0.20%	0.073^a^
26	If we offer an osteoporosis public seminar, would you like to attend?	82.40%	9.80%	0.2^a^
Knowledge score on osteoporosis (KOS)		69 ± 7.6	71 ± 8.4	<0.0001^b^

^a^Chi-squared *t*-test. ^b^Independent *t*-test.

**Table 4:** Binominal logistic regression for predicting adequate knowledge towards osteoporosis among the study sample.

Variables	Categories	Odds ratio	95% CI (lower-upper)	*P* value
Age range	18-30	Reference		0.193
	31-40	0.913	(0.741-1.126)	0.395
	Above 40	0.772	(0.583-1.022)	0.071

Social status	Single	Reference		0.094
	Married	0.802	(0.671-0.958)	0.015
	Divorced	0.859	(0.556-1.325)	0.491
	Widow	0.951	(0.643-1.407)	0.802

Weight	Under 50 kg	Reference		<0.0001
	50-60 kg	3.673	(2.617-5.155)	<0.0001
	61-80 kg	4.234	(3.010-5.956)	<0.0001
	Above 80 kg	9.170	(0.125-0.285)	<0.0001

Height	Under 150 cm	Reference		<0.0001
	150-160 cm	0.227	(0.165-0.311)	<0.0001
	161-170 cm	0.199	(0.144-0.276)	<0.0001
	Above 170 cm	0.189	(0.125-0.285)	<0.0001

Region	Rural region	Reference		<0.0001
	City	1.472	(1.258-1.723)	

Educational level	Illiterate	Reference		0.141
	Primary school	2.053	(1.106-3.813)	0.023
	Secondary school	1.557	(0.862-2.813)	0.143
	University stage or above	1.615	(0.908-2.873)	0.103

Economic level	Bad	Reference		<0.0001
	Moderate	0.781	(0.561-1.087)	0.143
	Good	0.775	(0.563-1.067)	0.118
	High	1.356	(0.919-1.999)	0.125

Menstrual status	Age of reproductive activity (existing menstrual cycle).	Reference		0.036
	Menopause period (menstrual cycle stopped)	1.381	(1.022-1.865)	

## Data Availability

The data are available from the corresponding author upon reasonable request.

## References

[1] Salari N., Darvishi N., Bartina Y. (2021). Global prevalence of osteoporosis among the world older adults: a comprehensive systematic review and meta-analysis. *Journal of Orthopaedic Surgery and Research*.

[2] Barnsley J., Buckland G., Chan P. E. (2021). Pathophysiology and treatment of osteoporosis: challenges for clinical practice in older people. *Aging Clinical and Experimental Research*.

[3] Salari N., Ghasemi H., Mohammadi L. (2021). The global prevalence of osteoporosis in the world: a comprehensive systematic review and meta-analysis. *Journal of Orthopaedic Surgery and Research*.

[4] Thuc S. C., Thi T. H. L., Thi M. T. N., Vinh H. D. (2021). Research article incidence rate and modifiable risk factors for osteoporosis in Vietnam. *Genetics and Molecular Research*.

[5] Somersalo A., Paloneva J., Kautiainen H., Lönnroos E., Heinänen M., Kiviranta I. (2014). Incidence of fractures requiring inpatient care. *Acta Orthopaedica*.

[6] Bakir M., Hammad K., Habil K. (2018). Bone mineral density in healthy Syrian women measured by dual energyX-ray absorptiometry. *Anthropological Review*.

[7] Pitzul K. B., Wodchis W. P., Kreder H. J., Carter M. W., Jaglal S. B. (2017). Discharge destination following hip fracture: comparative effectiveness and cost analyses. *Archives of Osteoporosis*.

[8] Tezbaşaran A. A. (1997). *Likert Tipi Ölçek Geliştirme Klavuzu*.

[9] Ungan M., Tümer M. (2001). Turkish women's knowledge of osteoporosis. *Family Practice*.

[10] Ware J. E. (1993). Measuring patients' views: the optimum outcome measure. *BMJ*.

[11] Ford M. A., Bass M., Zhao Y., Bai J. B., Zhao Y. (2011). Osteoporosis knowledge, self-efficacy, and beliefs among college students in the USA and China. *Journal of Osteoporosis*.

[12] Riaz M., Abid N., Patel J., Tariq M., Khan M. S., Zuberi L. (2008). Knowledge about osteoporosis among healthy women attending a tertiary care hospital. *The Journal of the Pakistan Medical Association*.

[13] von Hurst P. R., Wham C. A. (2007). Attitudes and knowledge about osteoporosis risk prevention: a survey of New Zealand women. *Public Health Nutrition*.

[14] Winzenberg T. M., Oldenburg B., Frendin S., Jones G. (2003). The design of a valid and reliable questionnaire to measure osteoporosis knowledge in women: the Osteoporosis Knowledge Assessment Tool (OKAT). *BMC Musculoskeletal Disorders*.

[15] Sayed-Hassan R. M., Bashour H. N. (2013). The reliability of the Arabic version of Osteoporosis Knowledge Assessment Tool (OKAT) and the osteoporosis health belief scale (OHBS). *BMC Research Notes*.

[16] Zhang Q., Cai W., Wang G., Shen X. (2020). Prevalence and contributing factors of osteoporosis in the elderly over 70 years old: an epidemiological study of several community health centers in Shanghai. *Annals of palliative medicine*.

[17] Chen P., Li Z., Hu Y. (2016). Prevalence of osteoporosis in China: a meta-analysis and systematic review. *BMC Public Health*.

[18] Foundation NO (2002). *America's bone health: the state of osteoporosis and low bone mass in our nation*.

[19] International Osteoporosis Foundation IFO (2022). Epidemiology of osteoporosis and fragility fractures. https://www.osteoporosis.foundation/facts-statistics/epidemiology-of-osteoporosis-and-fragility-fractures.

[20] Zamani M., Zamani V., Heidari B., Parsian H., Esmaeilnejad-Ganji S. M. (2018). Prevalence of osteoporosis with the World Health Organization diagnostic criteria in the Eastern Mediterranean region: a systematic review and meta-analysis. *Archives of Osteoporosis*.

[21] Gemalmaz A., Oge A. (2008). Knowledge and awareness about osteoporosis and its related factors among rural Turkish women. *Clinical Rheumatology*.

[22] Khan J. A., McGuigan F. E., Akesson K. E. (2019). Osteoporosis knowledge and awareness among university students in Saudi Arabia. *Archives of Osteoporosis*.

[23] Nguyen N. V., Dinh T. A., Ngo Q. V., Tran V. D., Breitkopf C. R. (2015). Awareness and knowledge of osteoporosis in Vietnamese women. *Asia-Pacific Journal of Public Health*.

[24] Puttapitakpong P., Chaikittisilpa S., Panyakhamlerd K., Nimnuan C., Jaisamrarn U., Taechakraichana N. (2014). Inter-correlation of knowledge, attitude, and osteoporosis preventive behaviors in women around the age of peak bone mass. *BMC Womens Health*.

[25] Abushaikha L., Omran S., Barrouq L. (2009). Osteoporosis knowledge among female school students in Jordan. *Eastern Mediterranean Health Journal*.

[26] Shakil A., Gimpel N. E., Rizvi H. (2010). Awareness and prevention of osteoporosis among South Asian women. *Journal of Community Health*.

[27] Sitati F. C., Obimbo M. M., Gichangi P. (2021). Knowledge and beliefs on osteoporosis among African postmenopausal women in a Kenyan semi-rural county of Kiambu. *Journal of Bone Metabolism*.

[28] Díaz-Correa L. M., Ramírez-García L. M., Castro-Santana L. E., Vilá L. M. (2014). Osteoporosis knowledge in patients with a first fragility fracture in Puerto Rico. *Boletín de la Asociación Médica de Puerto Rico*.

[29] Merle B., Haesebaert J., Bedouet A. (2019). Osteoporosis prevention: where are the barriers to improvement in French general practitioners? A qualitative study. *PLoS One*.

[30] Saw S. M., Hong C. Y., Lee J. (2003). Awareness and health beliefs of women towards osteoporosis. *Osteoporosis International*.

[31] Tan H. C., Seng J. J. B., Low L. L. (2021). Osteoporosis awareness among patients in Singapore (OASIS)-a community hospital perspective. *Archives of Osteoporosis*.

[32] Wang P., Abdin E., Shafie S., Chong S. A., Vaingankar J. A., Subramaniam M. (2019). Estimation of prevalence of osteoporosis using OSTA and its correlation with sociodemographic factors, disability and comorbidities. *International Journal of Environmental Research and Public Health*.

[33] Yeam C. T., Chia S., Tan H. C. C., Kwan Y. H., Fong W., Seng J. J. B. (2018). A systematic review of factors affecting medication adherence among patients with osteoporosis. *Osteoporosis International*.

[34] El Hage C., Hallit S., Akel M., Dagher E. (2019). Osteoporosis awareness and health beliefs among Lebanese women aged 40 years and above. *Osteoporosis International*.

[35] Francis J., Toh L. S., Sellappans R., Loo J. S. E. (2021). Awareness of osteoporosis risk assessment tools and screening recommendations among community pharmacists in Malaysia. *International Journal of Clinical Pharmacy*.

[36] Leslie W. D., Anderson W. A., Metge C. J., Manness L. J., Maximizing Osteoporosis Management in Manitoba Steering Committee (2007). Clinical risk factors for fracture in postmenopausal Canadian women: a population-based prevalence study. *Bone*.

[37] Hyassat D., Alyan T., Jaddou H., Ajlouni K. M. (2017). Prevalence and risk factors of osteoporosis among Jordanian postmenopausal women attending the national center for diabetes, endocrinology and genetics in Jordan. *BioResearch open access*.

